# Remarkable Differences in Calcification between the Primary Tumor and Metastatic Lymph Nodes in a Patient with ALK-Positive Non-Small-Cell Lung Cancer

**DOI:** 10.1155/2022/1160000

**Published:** 2022-02-26

**Authors:** Keeya Sunata, Tetsuo Tani, Takahiko Ui, Hidehiro Irie, Yohei Funatsu, Hidefumi Koh

**Affiliations:** ^1^Division of Pulmonary Medicine, Department of Internal Medicine, Tachikawa Hospital, Tokyo, Japan; ^2^Division of Pulmonary Medicine, Department of Medicine, Keio University School of Medicine, Tokyo, Japan; ^3^Department of Medical Oncology, Dana-Farber Cancer Institute, Boston, USA

## Abstract

Calcified bilateral mediastinal lymph nodes are not common in malignant tumors. A 51-year-old woman presented to our hospital with a 20 mm nodule in the lower left lobe of the lung and extensive calcification in the bilateral mediastinal lymph nodes. Computed tomography indicated no calcification of the primary lesion. Immunohistochemical staining and fluorescent in situ hybridization detected an anaplastic lymphoma kinase (ALK) fusion. Treatment with alectinib, an ALK inhibitor, led to a significant reduction in tumor size and calcification in the lymph nodes. This case shows that different degrees of calcification can be associated with malignant tumors and may be reversible in some cases.

## 1. Introduction

Lung cancer remains a deadly disease in spite of the recent development of treatments [1]. The treatment strategy for patients with lung cancer is based on the tumor stage. For early-stage lung cancer, the main goal is to achieve radical cure through surgical complete resection, and for advanced-stage lung cancer, the goal is to prolong the survival prognosis by administering various chemotherapies, molecular targeted drugs (i.e., EGFR inhibitors or ALK inhibitors), and immune checkpoint inhibitors [2].

During the process of the staging, the decision of presence or absence of distant metastasis and metastatic lymph nodes is crucial. Especially, lymph node metastasis is mainly determined by pathological or imaging findings. Secondary imaging findings other than lymph node swelling are also useful in the differential diagnosis. Lymph nodes with calcification are considered characteristic of benign diseases. They are particularly common in granulomatous diseases, such as sarcoidosis and tuberculosis. In addition, symmetrical and diffuse calcifications suggest a benign disease; on the other hand, multiple and stippled calcifications suggest a metastatic malignant tumor [3].

In advanced stage non-small-cell lung cancer (NSCLC), treatment options differ greatly depending on the presence of driver oncogenes. Recent developments in molecular diagnosis have enabled the detection of many driver genes such as EGFR or ALK alteration in NSCLC [2]. ALK fusions are found in approximately 5% of NSCLCs, and ALK inhibitors have shown high efficacy in these patients [4].

Here, we present a case of NSCLC in which no calcification was found in the primary tumor on imaging, but marked calcification was found in the mediastinal lymph node metastases, and the calcification almost disappeared after administration of the ALK inhibitor alectinib.

## 2. Case Presentation

A 51-year-old woman presented to our hospital with an abnormal shadow on chest radiography. She had no symptoms, no medical history, and no smoking habit. On physical examination, there was no fever, skin rash, or superficial lymphadenopathy.

**Figure 1:**
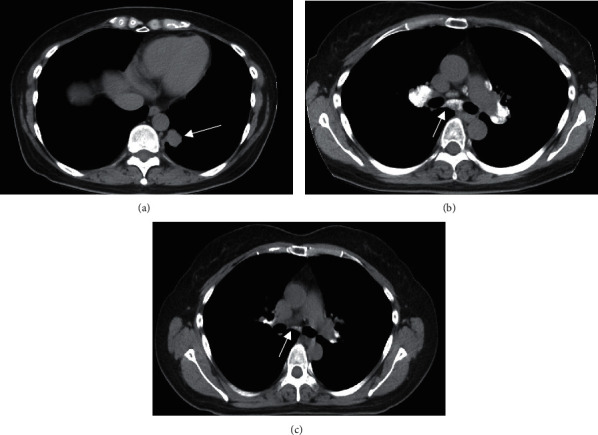
CT scan showing the primary lung tumor (a), calcification of the mediastinal lymph nodes before the administration of alectinib (b), and the lymph nodes 4 months after the administration of alectinib (c). White arrow showing subcarinal lymph nodes (b, c). CT: computed tomography.

**Figure 2:**
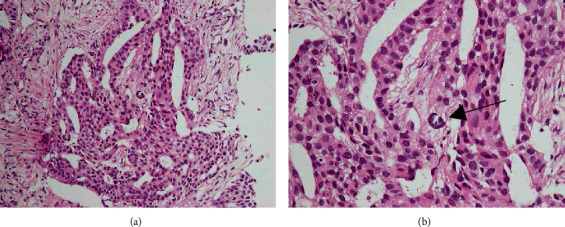
H&E of primary lung tumor (a) and black arrow showing psammoma body (b).

Laboratory results were as follows: Sialyl Lewis X-i antigen (SLX) 130 U/L (normal range≦38 U/L); calcium and angiotensin-converting enzyme (ACE) normal range; and T-SPOT, negative for the detection of Mycobacterium tuberculosis. A computed tomography (CT) scan revealed a 20 mm nodule in the lower left lobe (Figure 1(a)) and enlarged mediastinal lymph nodes with extensive calcification (Figure 1(b)). The calcification of the lymph nodes was remarkable, symmetrical, and homogeneous. However, CT indicated no calcification of the primary lesion. Brain metastases were detected using magnetic resonance imaging. Subcutaneous CT-guided biopsy of the primary tumor was performed, and the histological findings showed lung adenocarcinoma (Figure 2(a)) and psammoma bodies, which are round, microscopic calcified structures (Figure 2(b)) [5]. The patient was diagnosed with stage IV lung adenocarcinoma. Immunohistochemical staining and fluorescent in situ hybridization detected an ALK fusion, but real-time PCR did not detect an EGFR mutation. After administration of alectinib 600 mg/day, an ALK inhibitor, the calcified lymph nodes, primary tumor, and brain metastases were remarkably reduced. The treatment not only reduced the size of involved lymph nodes but also decreased the degree of calcification (short axis: 12.2 mm to 4.1 mm, and Hounsfield units 175.8 to 0.2 in subcarinal lymph nodes) (Figures 1(b) and 1(c)). There have been no signs of relapse after the administration of alectinib for 1 year.

## 3. Discussion

We report a case of remarkable lymph node calcification in a patient with ALK-positive lung cancer followed by almost complete clearance of calcification with alectinib.

Calcified lymph nodes are often observed in granulomatous diseases, such as tuberculosis, sarcoidosis, and amyloidosis; they are rarely observed in malignant tumors. Unterman and Reingold reported that approximately 6% of primary lung cancers are accompanied with calcification on CT scans and the frequency of calcification is particularly high in adenocarcinoma [6, 7]. Calcification in lung carcinoma is due to tumor necrosis, preexisting calcification, or secretions from the tumor, such as mucin [8]. Mahoney et al. reported that calcification caused by tumor necrosis is more common in small cell lung carcinoma, whereas preexisting calcification is more common in squamous cell carcinoma, and tumor secretions are more common in adenocarcinoma [7]. Tumor secretions, such as mucin, cause calcification and induce the formation of psammoma bodies. In this case, the patient had ALK-positive lung adenocarcinoma. ALK-positive lung cancer is often reported to be a mucin-producing lung cancer, such as signet ring cell carcinoma [9]. This case suggests that ALK-positive lung cancer may be more likely to be accompanied by calcification. Although previous reports suggest an association between EGFR mutations and calcification [10, 11], EGFR mutations were not detected in our case, and this is the first report suggesting an association between an ALK mutation and calcification.

In the case, there was a remarkable difference in the degree of calcification between the primary tumor and the metastatic lymph nodes. In addition, lymph node calcification was symmetrical and diffuse. These findings may lead to diagnostic difficulties. The patient had psammoma bodies in the primary tumor and brain metastases and was treated with alectinib, which resulted in marked lymph node shrinkage, leading to the diagnosis of lymph node metastasis. The cause of the difference of calcification between the primary tumor and lymph nodes is still unclear. However, the high frequency of lymph nodes in tuberculosis may indicate that immune cell accumulation or the surrounding environment in lymph nodes is preferable for calcification.

To the best of our knowledge, this is the first report to show that cancer-associated calcification is reversible with the administration of drugs and that it reflects disease activity. After the diagnosis of stage IV lung adenocarcinoma, we administered alectinib. Alectinib is an ALK tyrosine kinase inhibitor that has high efficacy for patients with ALK fusion and is administered as a first-line treatment for advanced-stage ALK-positive lung cancer. In this case, alectinib treatment resulted in almost complete disappearance of calcification and shrinkage of the lesion, and the effect persisted for more than a year.

In conclusion, calcification is commonly associated with benign diseases, but it can also be associated with malignant tumors. Additionally, there may be a remarkable difference in the degree of calcification between primary and metastatic lesions, and calcification associated with tumor cells may be reversible in some cases.
